# Numerical Analysis of the Freezing Behavior of Saturated Cementitious Materials with Different Amounts of Chloride

**DOI:** 10.3390/ma16196594

**Published:** 2023-10-08

**Authors:** Sekandar Zadran, Joško Ožbolt, Serena Gambarelli

**Affiliations:** Department of Mineral Building Materials, Materials Testing Institute (MPA), University of Stuttgart, 70569 Stuttgart, Germany; ozbolt@iwb.uni-stuttgart.de (J.O.); serena.gambarelli@mpa.uni-stuttgart.de (S.G.)

**Keywords:** cement paste, freezing, poromechanics, chloride concentration, 3D FE numerical model, microplane model, hygro-thermo-mechanical coupling

## Abstract

The freezing behavior of cement paste saturated with different chloride concentrations is investigated numerically with a coupled 3D hygro-thermo-mechanical FE analysis. The mathematical formulation of the freezing processes in the context of poromechanics takes into account the water (hydraulic) and ice pore pressures, as well as the distribution of heat (temperature) and strains. These quantities are calculated numerically based on three coupled differential equations, namely the static equilibrium equation and the equations for the transport of water and heat. The coupling between the mechanical (loading) and the non-mechanical processes (freezing) is performed using a staggered solution scheme. The proposed numerical approach is first validated using numerical and experimental studies from the literature dealing with two different cement pastes saturated with different amounts of chloride. The validated model is then used to investigate the effects of liquid water permeability, total porosity and pore size distribution on the freezing behavior of hardened cement paste. The results show that liquid water permeability has a strong effect on the pore pressure and deformation of the hardened cement paste. It is also shown that by decreasing the total porosity, the material becomes denser and contracts more as the temperature decreases, leading to a decrease in freezing strain. The results of this paper will provide important findings for the development of a simplified engineering model to investigate the mechanism that leads to freeze–thaw salt-induced damage to concrete structures in the framework of the DFG-funded research project.

## 1. Introduction

Frost damage is one of the most important factors affecting the durability of cementitious materials in regions with cold climates. Over the years, many theories have been presented to describe this complex phenomenon [1]. According to Powers, the increase in hydraulic pressure due to the increase in the volume of water during the transformation to ice leads to internal stresses in the pores, which cause the damage of cementitious materials [2]. During frost action, after the conversion of water to ice, there is a 9% increase in volume with subsequent water expulsion from the freezing pores to the unfrozen pores. However, Scherer [3] emphasized that the crystallization pressure of the ice and not the hydraulic pressure should be the predominant source of stress during freezing.

Damage due to frost action can be categorized as internal cracking and surface scaling [4]. Internal cracking leads to a lower material modulus of elasticity, a drop in tensile strength and an increase in porosity and permeability [5]. Internal damage affects the concrete mass and is characterized by significant microcracking of the cement paste and weakening of the bond between the solid matrix and particles. Under freeze–thaw cycles, calcium-silicate-hydrate (C-S-H) remains stable; however, portlandite (Ca(OH)_2_) and sulfoaluminates are partly dissolved and recrystallized in the air voids of the cement paste as fibrous secondary hydrates after repeated freeze–thaw cycles [6]. Surface scaling, which is most commonly associated with the presence of solutes, results in material surface loss at a solute concentration of roughly 3% [7]. Due to surface scaling, concrete particles are detached from the surface in the order of a few millimeters, and this is usually localized while the surrounding zone remains in good condition. Surface weathering, in extreme cases, can result in the loosening of the aggregates due to the loss of paste and leads to a gradual reduction in the strength of the structure [8].

The freezing behavior of cement-based materials under fully saturated condition has been experimentally investigated by a number of researchers. Zeng et al. [9] analyzed both numerically and experimentally the behavior of two cement pastes saturated with different amounts of chloride concentrations. Based on their experimental results, the ice saturation degree is influenced by both porosity and pore connectivity. The freezing strains during the first cooling phase obtained from the numerical simulations of the poroelastic model show a good agreement with the measured strains. To experimentally investigate the freeze–thaw induced damage and changes in the pore structure of cement mortar, a recent study was carried out by Wang et al. [10] using low-field NMR. Several mix proportions of mortar were used by changing water to cement ratio, amount of cement and fine aggregate. The findings of the experiments show mass loss and relative modulus of elasticity of the tested specimens following certain freeze–thaw cycles, both of which are important criteria for determining the extent of freeze–thaw damage of the specimens. Additionally, to establish the connection between freeze–thaw damage and pore structure degradation, the complete deterioration of mortar specimens was also observed. Zhang et al. [11] performed experimental study on the mechanical properties and pore structure degradation of concrete under freeze–thaw cycles with two different water to cement ratios, 0.45 and 0.55. Each freeze–thaw cycle lasted 3–4 h with a maximum number of 200 cycles. The experimental data from NMR showed that the number of freeze–thaw cycles gradually increased the porosity of concrete in addition to the amount of meso- and macro-pores. Additionally, the test findings demonstrate that as the number of freeze–thaw cycles increases, concrete’s compressive strength, flexural strength and splitting tensile strength decrease.

The advancement of computer simulation technology and the in-depth study of the coupling between fracture mechanics and thermodynamics have led to a gradual shift from complicated FTC experiments to numerical simulations. This shift has significantly shortened experimental time and expenses. Numerical simulation in FTCs involves the simulation of computer models for concrete [12]. Numerical methods are valuable tools in assessing the behavior of concrete under freeze and thaw conditions, especially in cold climates where the durability of concrete structures is a significant concern. It is important to note that the application of freeze–thaw numerical modeling is critical for designing and maintaining pavements that can withstand in regions with cold climates and temperature variations. These simulations help optimize material selection, design thickness and structural integrity, leading to longer-lasting and more cost-effective pavements. The engineering application of numerical methods can also be related to sustainable recycling techniques in pavement construction and maintenance, particularly in the context of using recycled materials in road infrastructures [13,14].

Several models are developed over the years to predict the thermo-mechanical behavior of porous building materials such as cement paste subjected to frost action. Powers introduced the well-known hydraulic and osmotic pressure theories, which formed the basic theory of frost damage [15,16]. A mathematical model based on the pore size distribution and desorption and absorption isotherms for concrete below and above 0 °C was proposed by Bažant et al. [17]. Based on poromechanics and local thermodynamic equilibrium conditions between various phases, Zuber et al. [18,19] presented a numerical model through implementing into a finite element (FE) code to predict the behavior of fully saturated cement-based materials subjected to freezing temperatures. The FE model proposed by Zuber et al. was used in [19] to predict the volume instability of hydrated cement systems upon freezing. Yang et al. [20] proposed a micromechanical model to simulate the expansion of cement paste, which considers the thermal dilation of the matrix and pressure in the pore space. Evolution of frost damage in a fully saturated porous material was investigated by Koniorczyk [21] under variable hygro-thermal conditions.

Due to significant advances in computer technologies, computational methods to explicitly incorporate freeze–thaw mechanisms have been recently developed. In a recent study conducted by Jin et al. [22], a quantitative analysis of the damage caused by freeze–thaw cycles in concrete was conducted. The concrete’s ability to withstand freeze–thaw conditions was assessed by employing a damage model that considers changes in the microscale pore structure. This research underlines the significance of adopting a multiscale approach when studying freeze–thaw damage. Based on the HYMOSTRUC3D hydration model, Liu et al. [23] generated virtual cement pastes with integrated crystallization pressure due to ice formation. This method enables the visualization of cracks and ice in the cement paste. However, the model is mainly based on the crystallization pressure of ice rather than the hydraulic pressure model, which was developed by Powers [11,12]. Gan et al. [24] investigated the changes in the pore structure of the concrete under freeze–thaw cycles using a three-dimensional mesonumerical model. The model is based on the porosity swelling theory, which takes into account the porosity expansion due to cyclic freeze–thaw attacks. A good agreement was observed between the numerical results from the model and the experimental ones in terms of freeze–thaw damage and compressive strength reduction of the material. To quantitatively study the mechanism of chloride diffusion under FTCs, a 2D three-phase mesoscopic numerical model on chloride diffusion in concrete subjected to freeze–thaw attack was proposed by Jiang et al. [25]. By using a time-dependent variable called porosity, which considers how freeze–thaw action influences the concrete pore structure and couples the freeze–thaw process with the chloride diffusion process at a temporal scale, this model takes into account the FTC-induced damage affected by chloride diffusion. Rhardane et al. [26] introduced a thermo-mechanical model on the basis of thermodynamic aspects and physical processes at the microscale to provide a quantitative assessment of the mechanisms caused by the freeze–thaw action in relation to the internal damage of cementitious materials. According to their numerical results, the existence of supercooling and delayed ice nucleation is by far the most detrimental source of deterioration. It was further mentioned that the degradation of material and reduction in Young’s modulus were considerably related to the water/cement ratio and the minimum freezing temperature during FTCs.

Despite proposed models by several researchers, the freezing process in porous media, such as concrete, still remains a very complex topic. The process involves interactions between heat transfer and moisture, phase change and deformation. Heat-moisture migration was studied by Olsen [27], whereas hygro-mechanical coupling has been studied by Zuber and Marchand [18]. However, only a few studies are available in the literature on the coupled thermo-hygro-mechanical behavior of concrete under freezing.

The primary objective of the current study is to numerically investigate the freezing behavior of two different cement pastes saturated with different chloride concentrations. The mathematical formulation of the model is based on the theory available in the literature [18]. The 3D coupled hygro-thermo-mechanical (HTM) model [28] implemented in the in-house FE code MASA [29] is used as a framework for the calculations. The mechanical part of the model is based on the microplane theory [30], suitable for the non-linear analysis of different materials, i.e., cement paste. However, in the present study, the material is considered linearly elastic. The coupling between the mechanical (loading) and non-mechanical processes (freezing) is ensured by using the staggered solution procedure. The model is first validated against a numerical and experimental study available in the literature [9]. The novelty of the validated model is mainly related to the parametric studies performed, where the effect of different pore size distributions and porosity values and their corresponding liquid water permeabilities are investigated on the freezing deformation of the cement pastes. It is worth mentioning that the proposed numerical approach will be used in the future to investigate the cyclic freeze–thaw behavior of cement paste and concrete by using a mesoscale modeling approach.

The paper is structured as follows. The mathematical formulation of the model, with related theoretical background, is provided in Section 2. Section 3 contains a description of the 3D FE model with the used input material parameters. To verify the model and its implementation into the 3D FE code, the numerical results are compared with the literature data. The influence of liquid water permeability, total porosity and pore size distribution on the freezing behavior of the PI cement paste is investigated in Section 4. The key findings are then reported in the conclusions (Section 5).

## 2. Mathematical Formulation of the Model

When a porous material is subjected to freezing, cryo-deformation occurs as a result of several combined effects: (i) the variation in density between the liquid water and the ice crystal; this variation in density leads to the expansion of the solid matrix surrounding the forming crystal and the displacement of some of the liquid water from the freezing sites toward the pores that are still filled with liquid water; (ii) the surface tension created between the various constituents, which ultimately determines the crystallization process in conjunction with the pore radius distribution; (iii) the drainage of liquid water displaced from the freezing sites toward the air pores; (iv) the cryo-suction process that drives liquid water toward the already frozen sites as the temperature continues to drop; (v) the thermomechanical coupling between the pressurized pore space and the surrounding solid matrix that determines the overall cryo-deformation [31].

The freezing behavior of cementitious materials is studied using a poromechanical approach. The material is considered a porous medium saturated with water and subjected to freezing. The thermodynamic laws involved are used to establish the constitutive equations for phase change, mass transfer and heat transfer. As a result, the pore pressure generated by freezing is converted into a macroscopic effective stress by a homogenization scheme. In the model proposed, the material is assumed to be fully saturated and linearly elastic. The governing variables are the pressure in the water fluid (*p_w_*), the temperature (*T*) and the strains (*ε*). The mathematical formulation of the freezing of a porous continuum is, in principle, the same as that presented by Zuber and Marchand [18]. The model is extended here with respect to the influence of chloride content on the freezing mechanism of porous materials.

### 2.1. Continuity Equation

**Figure 1:**
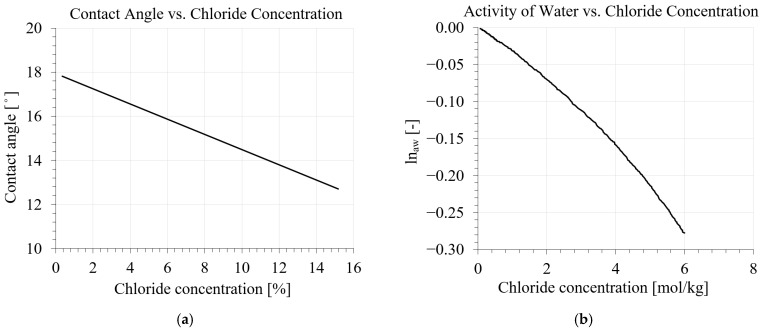
Effect of chloride concentration on the equilibrium pore radius: (**a**) relationship between chloride concentration and contact angle by Lide [36]; (**b**) relationship between chloride concentration and activity of water by Lin [37].

In accordance with Darcy’s constitutive law and the mass balance of the porous media, accounting for the effect of the coupling between deformation, ice formation and heat and water migration, the constitutive equation for the pressure in the liquid water was formulated by Zuber and Marchand [18]:

(1) $$\beta {\dot{p}}_{w}=\nabla \cdot \left(\frac{D}{\eta }\nabla p_{w}\right)+S-b{\dot{\varepsilon }}_{v},$$

where *p_w_* = pressure in the liquid water; *D* = permeability (m^2^) of the porous media; *η* = fluid viscosity (Pa s); $\varepsilon_{v}$ = volumetric strain of the medium; *b* = Biot’s coefficient (dimensionless) and:

(2) $$\beta =\left(\frac{nS_{w}^{}}{K_{w}}+\frac{nS_{i}^{}}{K_{i}}+\frac{b-n}{K_{m}}\right),=\left(\frac{1}{\rho_{i}^{}\;}-\frac{1}{\rho_{w}^{}\;}\right){\dot{w}}_{i}+\bar{\alpha }\dot{T}-\frac{b-n}{K_{m}}\dot{X}-\frac{nS_{i}^{}}{K_{i}}\gamma \dot{\kappa },$$

(3) $$\bar{\alpha }=nS_{w}^{}\alpha_{w}+nS_{i}^{}\alpha_{i}+\left(b-n\right)\alpha_{0\;},$$

(4) $$R_{peq}=R_{eq}+\delta ,\;R_{eq}=\frac{2\tau \;cos\Theta }{\rho_{i}\;S_{f}\left|\theta \right|\;},\;\delta =1.97{\left|\theta \right|}^{-\frac{1}{3}},\;\theta =T_{f}-T_{0},$$

(5) $$\kappa =\frac{2}{R_{eq}},\;X=\frac{\tau }{n}\int_{\infty }^{R_{peq}}\left(\kappa -\frac{1}{r-\delta }\right)\frac{d\varphi }{dr}dr,\;\varphi \left(r\right)=\int_{r}^{\infty }\frac{d\varphi }{dr}dr,$$

in which *n* = total porosity of a given mixture (m^3^/m^3^); *r* = effective pore radius (m); $\varphi \left(r\right)$ = cumulative volume of pores with a radius greater than *r* (m^3^/m^3^); *T_f_ =* freezing temperature of pore water (K); *T*_0_ = normal freezing temperature of bulk water (273.15 K); *S_i_* and *S_w_* = proportions of the porosity filled with ice and liquid water, respectively (*S_i_* + *S_w_* = 1); *K_w_* and *K_i_* = compressibility modulus of water and ice (Pa), respectively; *K_m_* = compressibility modulus of the solid matrix (Pa); *ρ_i_* = mass density of ice (kg/m^3^); *ρ_w_* = mass density of water (kg/m^3^); *w_i_* = mass of frozen water (kg/m^3^); *X* = pressure, which is related to the presence of ice in the frozen pores (Pa); *κ* (curvature) accounts for the effects of the spherical interface between liquid water and ice (m^−1^) (note that 2 stays for cylindrical pores); $\alpha_{w}$ and $\alpha_{i}$ = volumetric thermal dilatation of water; and ice (K^−1^), respectively; $\alpha_{0}$ and $\bar{\alpha }$ = volumetric thermal dilatation of bulk skeleton and of the system (K^−1^), respectively; $R_{peq}\;$= equilibrium (critical) pore radius (nm); $R_{eq}$ = radius of curvature of the liquid/ice interface (nm); *δ* = thickness of the liquid-like layer between the pore ice surface and matrix that remains unfrozen (nm); τ = surface tension of ice/water interface (N/m): Θ = contact angle between the crystal and pore wall calculated from Figure 1a (degree); *S_f_* = fusion entropy density of ice (J/(K kg)).

#### 2.1.1. Equilibrium Pore Radius

The pore structure of a porous medium (cement paste) has a great influence on the frost resistance of the material [9]. During the cooling process, the water trapped in the pores can freeze at different temperatures depending on the pore size. The smaller the pore radius, the lower the temperature at which the ice forms. When the temperature drops below the freezing point of water (*T*_0_), ice crystals initially form in the larger pores. With a further decrease in temperature, the ice crystals also begin to form in the small pores. An important aspect is the surface interaction between the matrix and the pore water or pore ice, as it causes freezing depression.

Due to the surface contributions to the free energy, the freezing point reduces with decreasing pore size. The equilibrium (critical) pore radius $\left(R_{peq}\right)$ at which water in the pores starts freezing when the temperature *θ* (in degrees Celsius) is reached is calculated based on the well-known Gibbs–Thomson equation for pure water (Equation (4)). It represents the relationship between the equilibrium pore radius at a given temperature *T,* below which ice cannot be formed [32]. The presence of the water layer (*δ*) between the pore ice surface and matrix was experimentally proved and is calculated according to Equation (4) proposed by Fagerlund [33].

It is well known that as the concentration of chloride in water increases, the freezing temperature *T*_0_ decreases. This is also valid for pore water. The equilibrium pore radius $\left(R_{peq}\right)$ for water with different chloride concentrations at a given temperature is calculated as [34]:

(6) $$R_{peq}=R_{eq}+\delta ,\;R_{eq}=\frac{2\tau \;cos\Theta \;V_{c}}{RT_{f}\;ln_{aw}-S_{f}\;\left|\theta \right|\;},\;\delta =1.97{\left|\theta \right|}^{-\frac{1}{3}},\;\theta =T_{f}-T_{0},$$

where *R* = gas constant (J/(K mol)); $V_{c}$ = molar volume of ice (m^3^/mol), *ln_aw_* = activity of water in solution calculated from Figure 1b; *S_f_* = molar fusion entropy (J/(K mol)). Parameters related to the calculation of equilibrium pore radius are shown in Table 1.

The effect of chloride in the calculation of equilibrium pore radius is considered by including the contact angle and activity of water parameters, which are functions of salinity. The relationship between contact angle and chloride concentration (%) is shown in Figure 1a [36]. For a given molality, the value of the activity of water is calculated from the curve in Figure 1b, which was provided by Lin and Lee [37].

As can be seen from Figure 1a,b, the value of chloride concentration for the calculation of contact angle and activity of water is introduced as percentage and molality, respectively. The below relationship can be used to convert the concentration of chloride from percentage to molality:

(7) $$\mathrm{chloride}\;\mathrm{molality}=\frac{\mathrm{mass}\;\mathrm{of}\;\mathrm{chloride}\;\left(\frac{g}{\mathrm{kg}\;\mathrm{of}\;\mathrm{water}}\right)}{\mathrm{molar}\;\mathrm{mass}\;\mathrm{of}\;\mathrm{chloride}\;\left(\frac{g}{\mathrm{mol}}\right)}\left[\frac{\mathrm{mol}}{\mathrm{kg}\;\mathrm{of}\;\mathrm{water}}\right],$$

**Figure 2:**
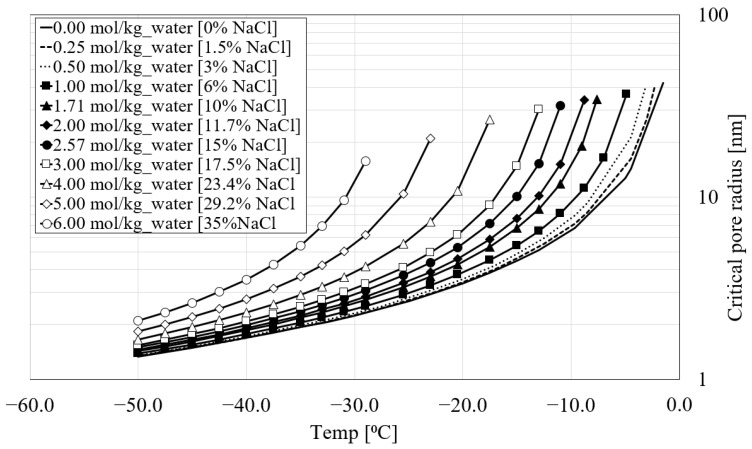
Evolution of equilibrium pore radius with varying chloride concentrations and temperatures.

According to Equation (4) for pure water and Equation (6) for water with different levels of chloride concentration, at a given temperature and molality, pores with an equilibrium pore radius larger than $R_{peq}$ should freeze, while those with a radius smaller than $R_{peq}$ should remain unfrozen. Based on these two equations, Figure 2 depicts the development of the equilibrium pore radius of the porous medium (cement paste) with respect to the varying chloride concentrations and temperatures. It can be seen that the freezing temperature decreases as the pore radius becomes smaller and the salinity of the water increases.

#### 2.1.2. Effective Pore Pressure

In Equation (1), *S* stands for the source of pressure. It consists of four terms. The first term denotes the pressure created by the formation of ice. The second term is related to the pressure generated during temperature changes due to the difference between the thermal expansions of various phases. The last two terms are related to the depression imposed on liquid water through the ice/water interface.

According to the homogenization approach proposed in [32], the effective pore pressure *p*^∗^ of the frozen continuum at the macroscale is calculated as follows:

(8) $$p^{*}=p_{w}+X,$$

where, as mentioned above, *X* (Equation (5)) is related to the pressure due to the presence of ice in the frozen pores, $p_{w}$ denotes liquid pressure. Note that $p^{*}$ (internal pore pressure) is the source of mechanical action and enters into the equilibrium equation.

### 2.2. Energy Conservation Equation

The differential equation for heat conduction can be written in the following form [32]:

(9) $$\rho C\dot{T}=\nabla \cdot \left(\lambda \;\nabla T\right)+L\;{\dot{w}}_{i},$$

$$\lambda =\frac{nS_{w}\lambda_{w}+nS_{i}\lambda_{i}+\lambda_{m}}{nS_{w}+nS_{i}+1}\;\;\;\;C=\frac{nS_{w}C_{w}+nS_{i}C_{i}+C_{m}}{nS_{w}+nS_{i}+1},$$

where *ρ* = density of the system (kg/m^3^); $\lambda_{}$*,*$\lambda_{w},\lambda_{i},\lambda_{m}$ = thermal conductivity of the system, water, ice and solid matrix, respectively (W/(m K)); $C_{},C_{w},C_{i},C_{m}$ = heat capacity of the system, water, ice and solid matrix, respectively (J/(kg K)); and *L* = latent heat of fusion of water (kJ/kg). The last term in Equation (9) describes a coupling between the heat transfer and the phase change and has a significant effect on freeze–thaw problems.

### 2.3. Momentum Equation

The governing equation for the mechanical behavior of a continuous body in case of static loading condition reads:

(10) $$\nabla \left(C_{m}\nabla u\right)-\nabla p_{T}-b\nabla p^{*}+f=0,$$

where $C_{m}$ = material stiffness tensor; *u* = displacement field; $p_{T}$ = volumetric stress due to free thermal strains of the porous material; *f* = specific volume load.

## 3. Verification of the Model: 3D Finite Element Analysis

To solve the above partial differential equations by using finite elements, the strong form of the formulation (Equations (1), (9) and (10)) is first rewritten into a weak form [38]. The weak form of the system of partial differential equations is based on the Galerkin weighted residual method. Subsequently, the model is implemented into a 3D FE code. The non-mechanical part of the problem (Equations (1)–(5)) is solved iteratively by using the direct integration method of implicit type [39]. To solve the mechanical part (Equation (10)), a Newton–Raphson iterative scheme is used. The coupling between the mechanical and non-mechanical parts of the model is performed by a continuous update of governing parameters during the incremental transient finite element analysis using a staggered solution scheme. The analysis is performed assuming a saturated continuum and linear elasticity.

### 3.1. Geometry and FE Discretization

The present numerical study is performed based on the experimental and numerical work of Zeng et al. [9] on the freezing behavior of cement paste saturated with different chloride concentrations. The cylindrical specimens have a diameter of 10 mm and a height of 100 mm. In their original work, two different cement pastes (0.5 and 0.3 *w*/*c* ratios) and six different chloride concentrations were investigated numerically and experimentally. The experimental results reported in [9] include four freeze–thaw cycles. The results, based on the poroelastic model proposed in [9], show fairly good agreement with the experimental tests for the first cooling phase.

In the present study, the cylinders with two cement pastes investigated in [9] were numerically analyzed to reproduce the freezing behavior of the material when saturated with different chloride concentrations. The water/cement ratio, boundary conditions and freezing rate were set as given in [9] (see Table 2). As in the experimental tests, cement pastes with water/cement ratios of 0.5 (PI) and 0.3 (PII) were investigated. The results from [9] were used to verify the numerical model.

**Figure 3:**
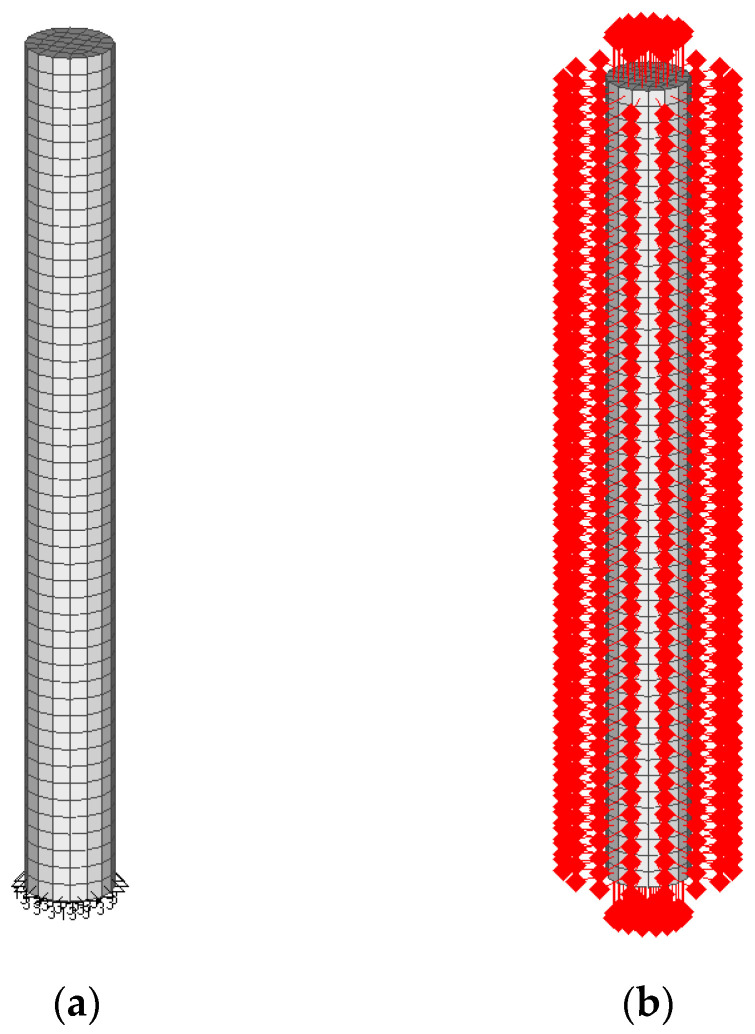
FE model: (**a**) mechanical boundary condition; (**b**) environmental boundary condition.

**Figure 4:**
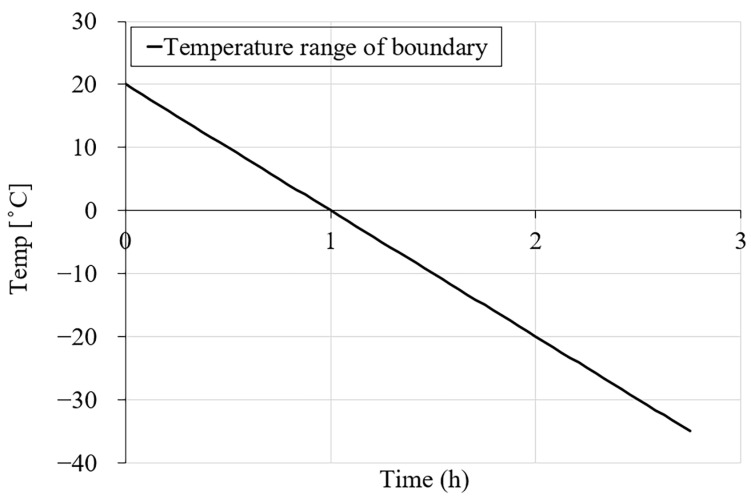
Temperature–time variation for the first cooling phase.

The FE discretization was performed using 1600 solid eight-node elements with an average element size of 2 mm. (Figure 3a). The mechanical and environmental boundary conditions of the FE model are shown in Figure 3a,b. Based on the information provided in [9], the specimens were kept vertically inside the environmental chamber with all free surfaces. Therefore, a simply supported constraint was applied to the bottom surface of the model (see Figure 3a). During the freezing test in [9], the temperature variation was applied through the environmental chamber to all surfaces of the specimens with the same freezing rate, as illustrated in Figure 4. The freezing rate for the first cooling phase was 20 °C/h, which lasted for 2.75 h. The hydraulic pore pressure at the surface of the specimen and the initial hydraulic pressure were set to zero, e.g., approximately atmospheric pressure (0.10 MPa).

**Table 2:** Two simulation cases.

Case	Water/Cement Ratio	Size (mm)	Chloride Concentration (%)	Freezing Rate and Temperature Range
1	0.5	10 × 100 cylinder	0, 1.5, 3, 6, 10, 15	Figure 4
2	0.3	10 × 100 cylinder	0, 1.5, 3, 6, 10, 15	Figure 4

As in [9], the environmental boundary conditions were applied to all external surfaces of the model as heat flux (Figure 3b). The time step for the numerical simulation was set to 150 s throughout the freezing phase. The first cooling phase of the simulation consists of 66-time steps corresponding to 2.75 h.

### 3.2. Material Parameters

The input parameters of the porous medium and solid skeleton for PI (PII) cement pastes are summarized in Table 3. Water/cement ratio, total porosity, compressibility modulus of the porous skeleton and compressibility modulus of the solid matrix were provided in [9]. Biot’s coefficient is calculated as follows:

(11) $$b=1-\frac{K_{0}}{K_{m}},$$

where *K*_0_ is the compressibility modulus of the porous skeleton and *K_m_* is the compressibility modulus of the solid matrix. The elastic modulus and liquid water permeability of PI and PII are calculated based on Equations (12) and (13), respectively. The Poisson’s ratio, thermal conductivity and heat capacity of the cement pastes are extracted from the literature.

The elastic modulus of PI and PII cement pastes listed in Table 3 was not directly provided in [9]; however, in the current model, it is calculated based on Equation (12), which sets the relationship between the compressibility modulus of the porous skeleton (*K*_0_) and Poisson’s ratio (*ν*).

(12) $$K_{0}=\frac{E}{3\left(1-2\nu \right)},$$

The parameter of liquid water permeability was also not provided in the poroelastic model from [9] because it was assumed that freezing can instantaneously occur to the entire sample due to its small size. Here, the value of the liquid water permeability for PI and PII is calculated based on the total porosity using the formula provided by Powers in [2]. The relationship from [2] is modified with one smaller order of magnitude such that the freezing strains best fit the experimental and numerical results in [9]. This modification is mainly due to the fact that the value of liquid water permeability is varying to a large extent, and it is difficult to find a fixed value for a given porosity and water-to-cement ratio. The experimental and numerical data on the value of liquid water permeability for cement pastes presented in [41] and other literature shows that this parameter varies from $10^{-23}\;m^{2}\;\mathrm{to}\;10^{-17}{\;m}^{2}$. This significant variation is caused by the selected experimental and numerical methods, the material’s pore structure and pore size distribution, as well as the level of saturation. The final relationship between liquid water permeability and porosity is taken as Equation (13):

(13) $$D=3.55\left(n^{3.6}\right)10^{-19}\left[m^{2}\right],$$

where *n* is the total porosity.

The thermal expansion coefficient values for two different cement pastes (PI and PII) were calculated based on the total porosity of the cement paste provided in [9] as Equation (14) [40]:

(14) $$\alpha_{d}=\alpha_{c}{\left(1-n\right)}^{2.66},$$

with $\alpha_{c}=26.98\times 10^{-6}\;{/}^{{}^{\circ}}C$

Table 4 presents the input material parameters for water and ice. These parameters are mainly extracted from [18,35].

### 3.3. Pore Size Distribution

**Figure 5:**
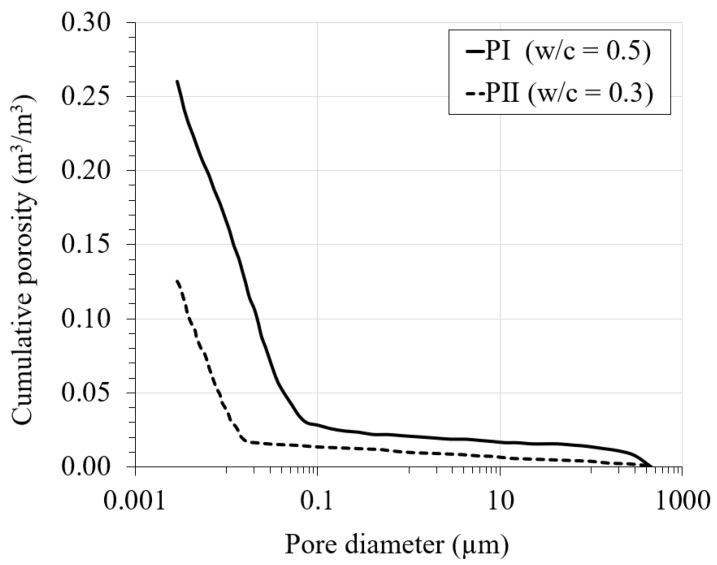
Pore size distribution of PI and PII cement pastes.

The pore size distribution (PSD) curves used in the model are taken from [9]. The curves were experimentally obtained for PI and PII cement pastes through the mercury intrusion porosimetry (MIP) method. In [9], the PSD curves are provided in terms of pore volume (mL/g) versus pore diameter (nm) for both PI and PII cement pastes, with the maximum values of intruded specific pore volume of 0.161 and 0.063 mL/g, respectively. Subsequently, the PSD curves from [9] were adapted by converting the pore volume on the y-axis into cumulative porosity simply by multiplying the pore volume by the apparent density of the cement pastes. The apparent density (including pores and skeletons) reported by the author of the paper for PI approximates 1.6 g/mL, while that of PII approximates 2 g/mL. Figure 5 shows the obtained pore size distribution curves.

### 3.4. Numerical Results and Discussion for PI Cement Paste (0.5 w/c Ratio)

#### 3.4.1. Freezing Strains

As previously discussed, Zeng et al. [9] performed experimental and numerical studies on two cement pastes with a water/cement ratio of 0.5 (PI) and 0.3 (PII), respectively. The authors analyzed the cylindrical specimen (height = 100 mm, diameter = 10 mm) saturated with different chloride concentrations: 0, 1.5, 3, 6, 10 and 15%. The goal of the study was to investigate the freezing behavior of saturated cement pastes during four freeze–thaw cycles. Using a special experimental setup and an LVDT stand, the average axial strain of the cylindrical specimens was experimentally measured in an undrained condition. The experimental results showed that the ice saturation degree was influenced by both porosity and pore connectivity of the two different pastes.

**Figure 6:**
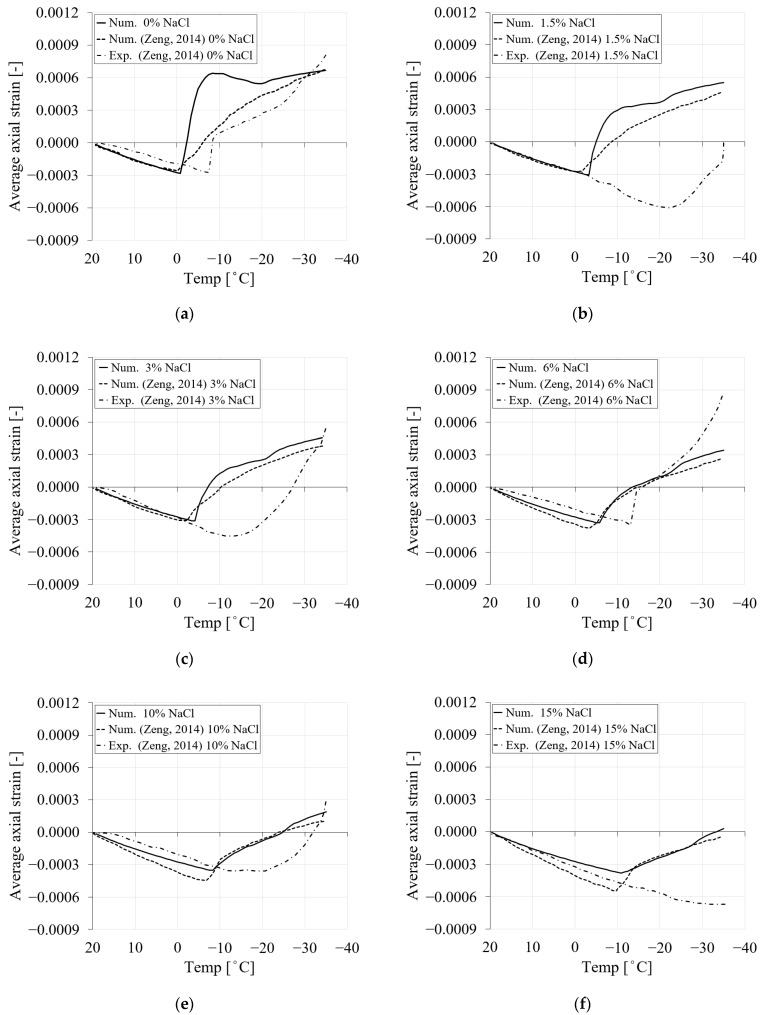
The comparison between the freezing strains of the current study and experimental and numerical results from Zeng [9] for PI cement paste during the first cooling phase: (**a**) pure water; (**b**) 1.5% NaCl; (**c**) 3% NaCl; (**d**) 6% NaCl; (**e**) 10% NaCl; (**f**) 15% NaCl.

Similar to the experimental and numerical work presented in [9], in the present study, two sets of simulations were conducted for 0.5 and 0.3 water/cement ratios and chloride concentrations of 0, 1.5, 3, 6, 10 and 15% for the first cooling phase ranging from +20 to −35 °C (Figure 4). The results for the 0.5 *w*/*c* ratio in terms of average axial strains for the first cooling phase are shown in Figure 6 and compared with the experimental and numerical curves reported in [9]. The results of the second set of simulations (0.3 *w*/*c* ratio) are provided in Appendix A.

As can be seen from Figure 6, the results obtained in the present numerical study agree well with the numerical results obtained in [9]. Comparing the numerical curves, a transition between the contraction phase and the expansion phase in terms of the average uniaxial strain can be observed in all cases. In particular, the transition point decreases as the amount of chloride increases. For instance, in Figure 6, the temperature at the transition point for 0% NaCl corresponds to −0.1 °C, while for 1.5% NaCl, the corresponding transition point temperature is −3.3 °C. The temperatures at the transition points for 3%, 6%, 10% and 15% NaCl concentrations are −4.2 °C, −5.8 °C, −8.3 °C and −10.8 °C, respectively. This is consistent with the equilibrium pore radius curves shown in Figure 2. Figure 6 demonstrates that by increasing chloride concentration, the freezing point of the solution is shifted, resulting in less freezing strain.

It should be noted that the experimental results for 1.5 and 3% chloride concentrations from [9] (Figure 6b,c) show a stronger contraction when the temperature drops below the freezing point of water. According to [9], this additional contraction below the freezing point of water could be due to the initial incomplete saturation of the samples. Some occluded voids or capillary pores may behave like entrained air voids because they are either partially saturated or even empty. As a result, ice can easily form on the pore walls, attracting water (solution) contained in adjacent smaller pores. Considering the possibility of a partially saturated state given in [9] for the cases of 1.5% and 3% chloride, no reasonable agreement can be found between the experimental and numerical results for these two cases. However, in the present model, the material is assumed to be fully saturated, so the results presented here show good agreement with the numerical results in [9], except for the case with pure water in Figure 6a. In this case, the freezing strains obtained show steeper behavior after the temperature drops below the freezing point of the water. This behavior can be attributed to the fact that a different formulation for calculating the equilibrium pore radius for pure water was used in the model than in the other cases with chloride.

#### 3.4.2. Pore Pressure

**Figure 7:**
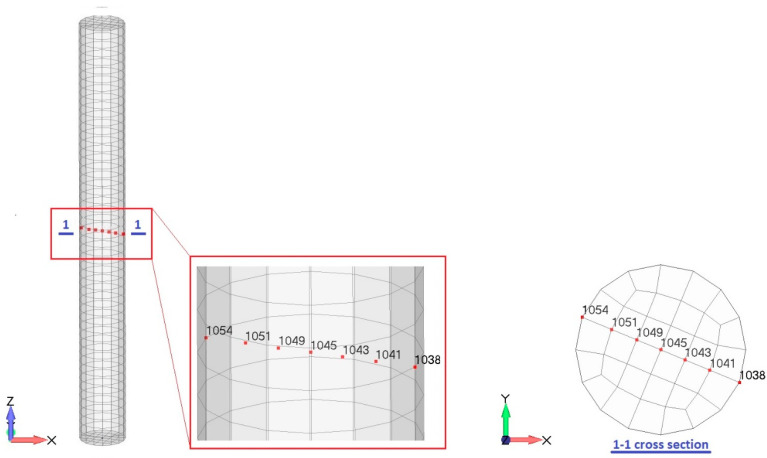
Arbitrary nodes for freezing-induced liquid, ice and pore pressure.

**Figure 8:**
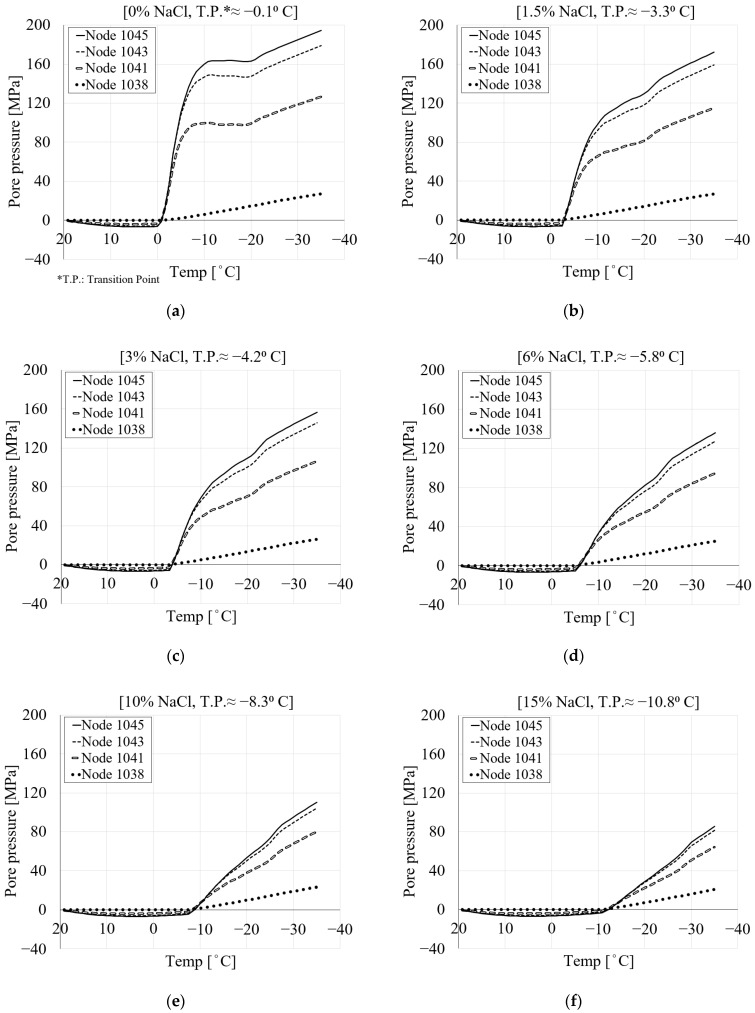
The comparison between the pore pressure of four selected nodes for PI cement paste during the first cooling phase with respect to temperature variation: (**a**) pure water; (**b**) 1.5% NaCl; (**c**) 3% NaCl; (**d**) 6% NaCl; (**e**) 10% NaCl; (**f**) 15% NaCl.

The pore pressure of porous media, as the sum of hydraulic and ice pressure, is directly related to the size and structure of the pores. In fully saturated conditions, an increase in the porosity of the material leads to more freezing, resulting in higher pore pressure and deformation. To observe the ice, liquid and pore pressure during the freezing process, seven nodes were selected across the mid-height of the specimen (see Figure 7). The evolution of the pore pressure with temperature is shown in Figure 8. Due to the symmetry, the results are shown only for nodes 1038, 1041, 1043 and 1045. Figure 8a shows a comparison between the pore pressure of the selected nodes with respect to their locations within the specimen for pure water. The results show that by moving towards the surface of the specimen, the pore pressure reduces. Based on the cooling regime, negative pore pressure as a result of the thermal contraction of the pore solution can be observed until the temperature reaches the freezing point of the pore solution. However, after the temperature goes below the freezing point of the solution, due to freezing inside the pores, positive pore pressure develops. This pore pressure is consistent with the freezing-induced expansion of the specimen.

Figure 8b–f show the pore pressure results of the same nodes for 1.5, 3, 6, 10 and 15% of chloride concentrations. It can be seen that by increasing the amount of chloride, the pore pressure decreases. This is mainly related to the fact that the freezing temperature of the solution decreases with increasing chloride concentration. Additionally, the transition point shifts towards the negative temperature as the amount of chloride increases (Figure 8). This agrees with the curves for the equilibrium pore radius shown in Figure 2. It should be noted that, due to the small size of the specimen, there is no time delay between the applied temperature and the freezing of the solution inside the pores. According to the authors of [9], their unpublished tests showed that the time lag for homogeneous temperature within the specimen was about 2 min.

**Figure 9:**
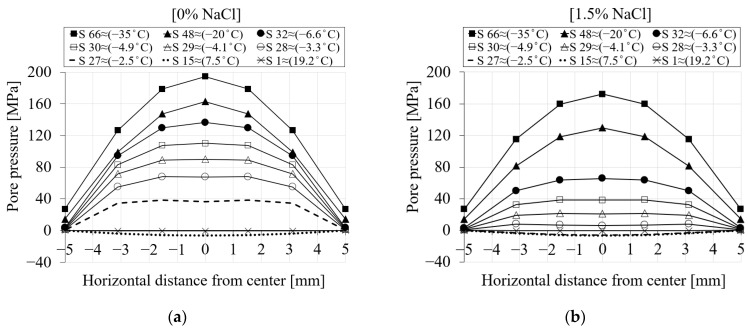

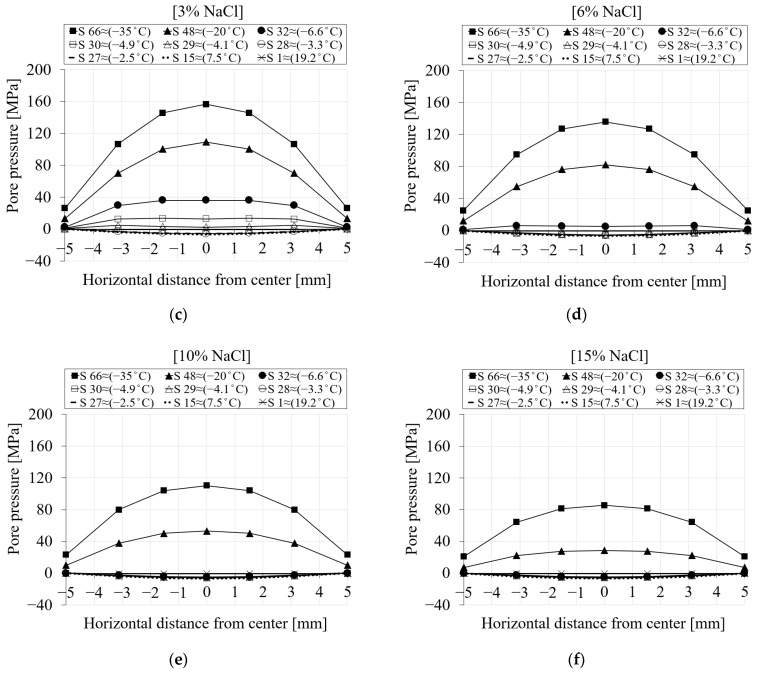
The comparison between the pore pressure of the seven selected nodes in terms of distance and time step for PI cement paste during the first cooling phase: (**a**) pure water; (**b**) 1.5% NaCl; (**c**) 3% NaCl; (**d**) 6% NaCl; (**e**) 10% NaCl; (**f**) 15% NaCl.

Figure 9 represents the spatial distribution of the pore pressure for the selected seven nodes (see Figure 7) during the first cooling phase. The pore pressure variation versus the distance of the nodes from the center of the specimen is shown for (i) six different chloride concentrations and (ii) nine time steps of the analysis. Each point on the curve corresponds to a selected node. The first step (S1 ≈ 19.2 °C) corresponds to the beginning of the cooling phase, while the last one (S66 ≈ −35 °C) refers to the end of the cooling phase. From Figure 9a for 0% chloride concentration, it can be observed that the pore pressure at step 1 has nearly a value of zero. As the temperature decreases towards the freezing point of the solution, negative pore pressure as a result of the contraction of the pore solution can be observed (S15 ≈ 7.5 °C). After the temperature goes below the freezing point of the solution in the pores, freezing takes place, which leads to a positive pore pressure (S27 ≈ −2.5 °C).

Figure 9b–f show the same results for 1.5, 3, 6, 10 and 15% chloride concentrations. It is clear from Figure 9b (1.5% NaCl) that the pore pressure of the corresponding time steps compared to 0% chloride concentration is smaller due to the delay in the freezing of solution inside the pores as a result of chloride concentration. For instance, the pore pressure corresponding to step 27 (≈−2.5 °C) for 0% chloride concentration (Figure 9a) has a positive value, indicating that the water inside the pores has already started freezing, while the pore pressure related to the same time step for 1.5% chloride concentration (Figure 9b) still shows contraction. A similar comparison can be carried out for the rest of the cases.

#### 3.4.3. Ice Pressure

**Figure 10:**
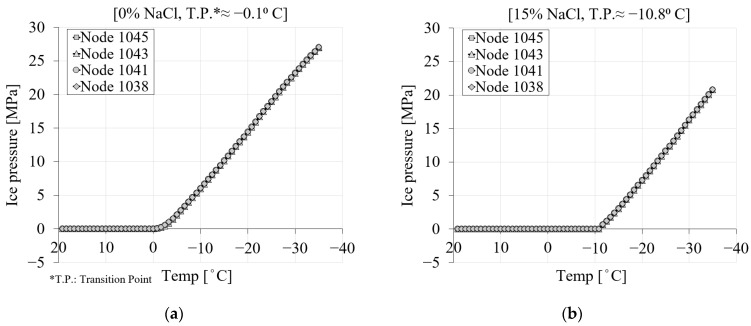
The comparison between the ice pressure of four selected nodes for PI cement paste during the first cooling phase with respect to temperature variation: (**a**) pure; (**b**) 15% NaCl.

Figure 10 shows the contribution of the ice pressure during the freezing process for two levels of chloride concentrations, namely, pure water and 15% chloride. It only depicts the ice pressure once the temperature goes below the freezing point of the solution. It is visible from the curves in Figure 10 that the ice pressure is zero until the solution inside the pores starts freezing. Depending on the concentration of chloride, the freezing point of the solution will decrease, and consequently, ice-induced pressure will be delayed. However, after the ice forms, regardless of the location of the nodes within the specimen, the ice pressure for a specific time step and chloride concentration becomes identical.

**Figure 11:**
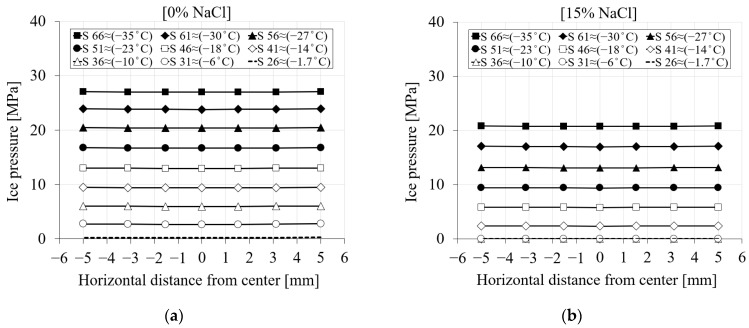
The comparison between the ice pressure of the seven selected nodes in terms of distance and time step for PI cement paste during the first cooling phase: (**a**) pure water; (**b**) 15% NaCl.

Figure 11 represents the ice pressure in terms of freezing time and distance of the nodes from the center of the specimen (see Figure 7) during the first cooling phase. Depending on the salinity of the solution inside the pores, the ice pressure starts generating only when the temperature goes below the freezing point of the solution. Since the ice pressure is not considerably affected by the quantity of chloride, Figure 11 only shows the ice pressure variation for two chloride concentration cases. Based on Figure 11a for 0% chloride concentration, the first line, which corresponds to step 26 (≈−1.7 °C), shows the onset of ice pressure. Figure 11b shows the ice pressure of the same time steps for a 15% chloride concentration. Depending on the high chloride concentration in this case, freezing of the pore solution starts somewhere around −11 °C. Therefore, the induced ice pressure has been delayed. The value of ice pressure for step 36 (≈−10 °C) in the case of a 15% chloride concentration is zero, while for the same time step, this value already reached ≈6 MPa for a 0% chloride concentration.

#### 3.4.4. Liquid [Hydraulic] Pressure

The liquid pressure inside the pores is an important source of freezing deformation in the porous medium. Liquid pressure is generated as a result of the viscous flow of water into the pore spaces in an undrained or low-permeability condition. According to [42], liquid pressure inside the pores may reach a magnitude of 150 MPa. Liquid pressure, together with ice pressure, forms pore pressure, which is the main source of freezing stress and deformation in porous materials.

**Figure 12:**
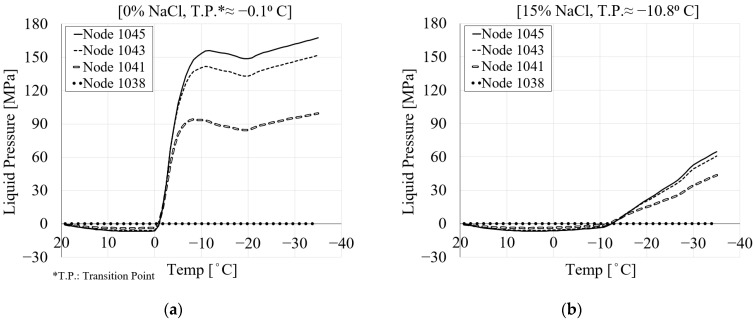
The comparison between the liquid pressure of four selected nodes for PI cement paste during the first cooling phase with respect to temperature variation: (**a**) pure; (**b**) 15% NaCl.

Figure 12 shows the liquid pressure of the selected four nodes for pure water and a 15% chloride concentration during the cooling phase. It is observed that the liquid pressure decreases from the center towards the specimen’s surface. The same behavior is also detected in the pore pressure variation with temperature (Figure 8). Before the temperature reaches the freezing point of the solution, thermal contraction takes place. Figure 12b shows that less liquid pressure is produced as the chloride concentration increases because the freezing process delays, which results in a large drop in liquid pressure.

**Figure 13:**
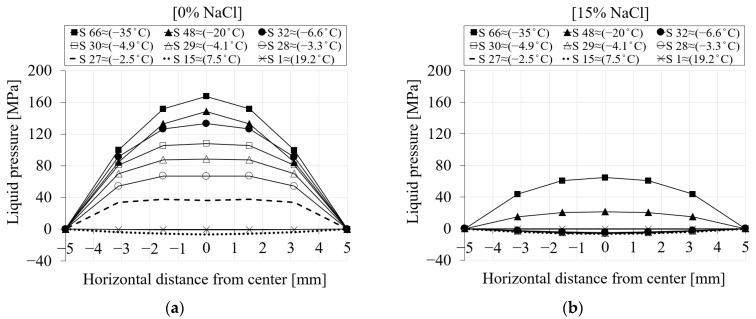
The comparison between the liquid pressure of the seven selected nodes in terms of distance and time step for PI cement paste during the first cooling phase: (**a**) pure water; (**b**) 15% NaCl.

To better visualize how the liquid pressure changes across the section of the specimen, Figure 13 shows the spatial representation of the liquid pressure for seven selected nodes during the cooling phases. Figure 13a shows the liquid pressure across the mid-height of the specimen for 0% chloride concentration. It is evident from Figure 13a that the liquid pressure at step 1 (≈19.2 °C) is almost zero. Negative liquid pressure due to the contraction of the pore solution may be seen when the temperature drops (S15 ≈ 7.5 °C). After the temperature goes below the freezing point of the solution in the pores (S27 ≈ −2.5 °C), positive liquid pressure starts developing. Liquid pressure increases as the temperature drops further. Compared to the maximum liquid pressure, which corresponds to the end of the cooling phase (S66 ≈ −35 °C), the main contribution of the liquid pressure comes from the freezing process a few steps below the freezing point of the solution (S32 and below). This is related to the fact that once all pores are frozen, the liquid pressure does not increase anymore and remains almost constant. However, it should be noted that the contribution of the ice pressure after the end of the liquid pressure cannot be neglected. Figure 13b shows the same representation of liquid pressure for a 15% chloride concentration. It is clear that when the chloride concentration increases, the freezing process takes longer and produces lower liquid pressure.

#### 3.4.5. Influence of the Freezing Point on the Freezing Process

To this end, the numerical results shown for PI cement pastes were based on the development of the equilibrium pore radius with varying chloride concentrations and temperatures, according to Figure 2. The average axial strain for PI cement paste reported in Figure 6 was in reasonable agreement with the numerical results in [9]. It is also clear from Figure 6 that the freezing point of the pore solution and the onset of the positive strain of the numerical study and the one reported in [9] for different chloride concentrations are almost the same. However, if a comparison is carried out between the numerical and experimental results in [9], it can be seen that for the experimental curves, there is a delay in the freezing process and freezing point of the pore solution. Considering that (i) experimentally it takes slightly longer for the freezing process to occur after the temperature (cooling) is applied and (ii) the degree of saturation of the pores affects the freezing-induced strains, the freezing points of the pore solution, as indicated in Figure 2 for the calculation of the equilibrium pore radius, were modified in the model to correspond to the points observed in the experiments [9].

**Figure 14:**
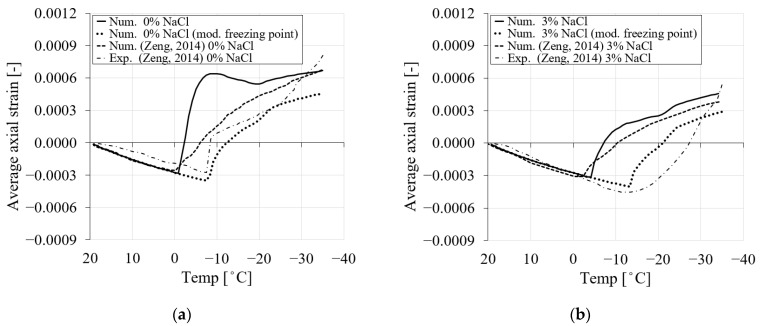
The comparison between the average axial strain of the modified and original freezing points of the present model and the numerical and experimental results from Zeng [9] in terms of freezing temperature for PI cement paste during the first cooling phase: (**a**) 0% NaCl; (**b**) 3% NaCl.

Figure 14 shows the average axial strain of PI cement paste for 0% and 3% chloride concentrations with the original and modified freezing points of the pore solution and their comparison with the results from [9]. The dotted line shows the average axial strain of the current study, where the freezing point of the solution was controlled based on the equilibrium pore radius in Figure 2. However, the solid line shows the same results with the modified freezing point, according to the experimental results [9]. It can be seen that a delay in the freezing process of the pore solution has a significant influence on the resulting average axial strain (Figure 14). The new numerical results for two chloride cases show very good agreement with the experimental results from [9].

**Figure 15:**
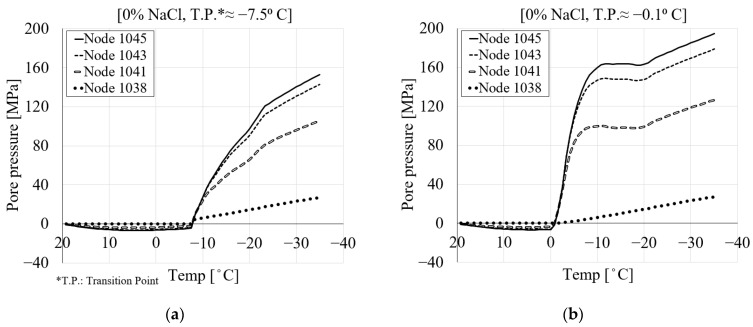
The comparison between the pore pressure of the modified and original freezing points of the present model in terms of freezing temperature for PI cement paste during the first cooling phase: (**a**) freezing point −9 °C and (**b**) freezing point −0.1 °C.

To investigate the effect of the delayed freezing temperature on the pore pressure of PI cement paste, Figure 15 shows the pore pressure for 0% chloride concentration, obtained with two different freezing points, i.e., −9 °C in Figure 15a and −0.1 °C in Figure 15b. The results are shown for the four nodes as previously selected (Figure 7). It is obvious from Figure 15a that by decreasing the freezing point of the pore solution, the freezing process delays and consequently results in lower pore pressure, which is the main source of deformation. Figure 15a also indicates that as the freezing point of the solution is reduced, the transition point also decreases accordingly, i.e., −7.5 °C.

**Figure 16:**
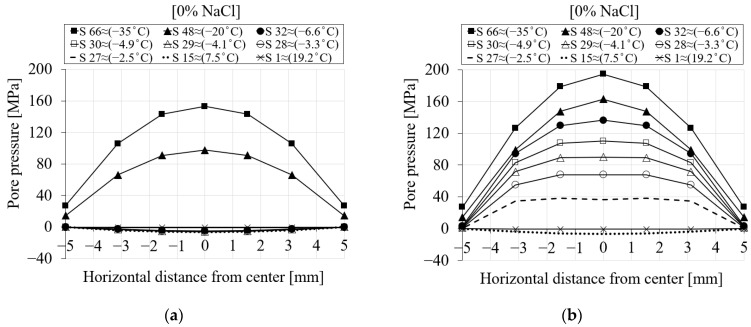
The comparison between the pore pressure results of the modified and original freezing points of the present model in terms of distance for PI cement paste during the first cooling phase: (**a**) freezing point −9 °C and (**b**) freezing point −0.1 °C.

To better clarify how the pore pressure distribution across the section is affected by the delay in the freezing process, a spatial representation of the results is shown in Figure 16 for 0% chloride concentration and two different freezing points, i.e., −9 °C in Figure 16a and −0.1 °C in Figure 16b. Based on the results in Figure 16b, positive pore pressure starts to generate approximately at step 27 (≈−2.5 °C), while for the same time step in Figure 16a, there is still contraction. This clearly indicates that by decreasing the freezing point of the pore solution, the freezing process is delayed. The same comparison could be carried out for all time steps.

## 4. Parametric Study

After the validation of the 3D FE model, a parametric study was performed on PI cement paste to investigate the influence of liquid water permeability, total porosity and pore size distribution on the average axial strain and generated ice and pore pressure for the first cooling phase. As in the previous simulations, all cases from 0 to 15% chloride concentration were analyzed. The parametric study was performed by varying (1) the liquid water permeability and (2) the porosity with corresponding pore size distribution and liquid water permeability (see Table 5). In the first case, only the effect of liquid water permeability was investigated, keeping all the other parameters, including porosity and pore size distribution, unchanged. Even though these parameters depend on each other, the aim here was to show how only a variation in the liquid water permeability can affect the material’s response in terms of pore pressure and deformation. Six values of permeability for liquid water were selected, three of which were above and three below the reference value (Table 5).

**Figure 17:**
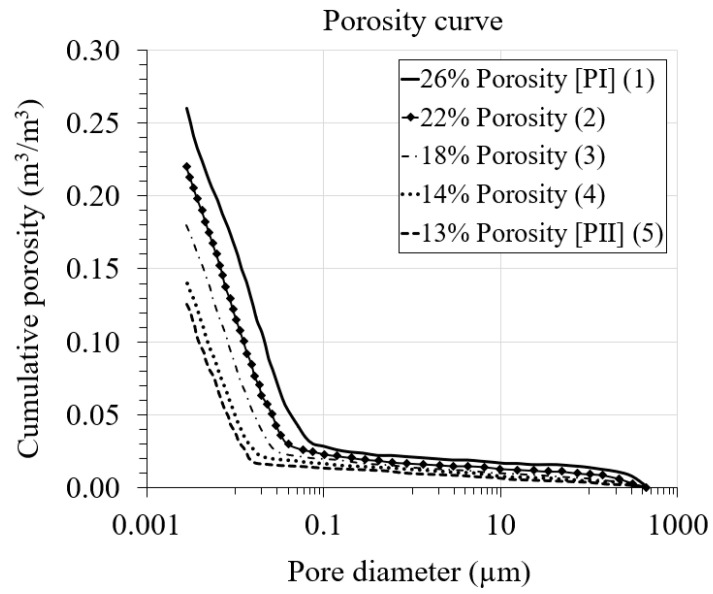
Pore size distribution for different porosity cases.

For the second case, considering simultaneously the effects of liquid water permeability, total porosity and pore size distribution, three values of total porosity smaller than the reference value were selected (Table 5). The corresponding pore size distribution curves based on the selected total porosity values were derived from the original porosity curve for PI cement paste in [9] and shown in Figure 17. The liquid water permeability value for each total porosity was calculated from Equation (13) and reported in Table 5. It is worth noting that higher porosity values were not chosen for this case compared to the reference value, since the porosity range for cement paste is generally between 10 and 25%.

**Table 5:** Parametric study values for PI cement paste.

**Case 1**	**Liquid Water Permeability (m^2^)** **(Ref. Value 2.78 × 10^−21^ m^2^)**	**Total Porosity**	**Pore Size Distribution**
	6.95 × 10^−22^ (–25% of Ref. value)	0.26	(1) in Figure 17
	3.48 × 10^−21^(+25% of Ref. value)	0.26	(1) in Figure 17
	1.39 × 10^−21^ (–50% of Ref. value)	0.26	(1) in Figure 17
	4.17 × 10^−21^ (+50% of Ref. value)	0.26	(1) in Figure 17
	2.09 × 10^−21^ (–75% of Ref. value)	0.26	(1) in Figure 17
	4.87 × 10^−21^ (+75% of Ref. value)	0.26	(1) in Figure 17
**Case 2**	**Total Porosity** **(Ref. Value 0.26)**	**Liquid Water Permeability (m^2^)**	**Pore Size Distribution**
	0.22 (85% of Ref. value)	1.52 × 10^−21^	(2) in Figure 17
	0.18 (70% of Ref. value)	7.40 × 10^−22^	(3) in Figure 17
	0.14 (55% of Ref. value)	2.99 × 10^−22^	(4) in Figure 17

### 4.1. Liquid Water Permeability

**Figure 18:**
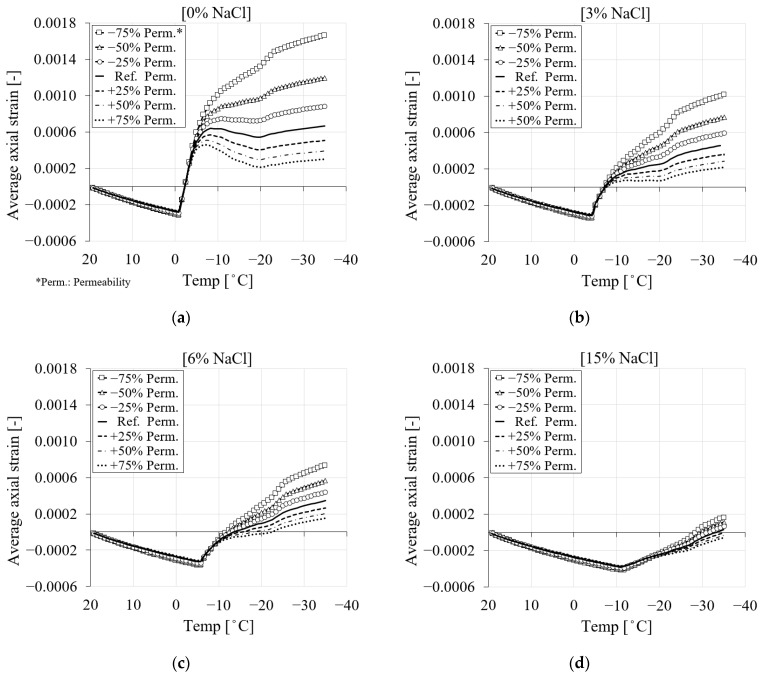
The comparison between the freezing strains of the reference liquid water permeability and six selected values based on Table 5 for PI cement paste during the first cooling phase: (**a**) pure water; (**b**) 3% NaCl; (**c**) 6% NaCl; (**d**) 15% NaCl.

In this series of studies, the influence of liquid water permeability compared to the calibrated (reference) value on the strain development and pore pressure of PI cement paste is investigated. The other material properties, including elastic modulus and boundary conditions, are kept unchanged. The numerical results of the simulations for four chloride concentrations (0, 3, 6 and 15%) in relation to the selected values of liquid water permeability in terms of average axial strain and freezing temperature are presented in Figure 18. Figure 18a shows that the reduced permeability of liquid water results in higher elongation in the case of pure water. This is due to the fact that, as a result of the decreased permeability, less water migrates through the capillary pores, causing more freezing to occur and consequently higher freezing strains. However, the increase in permeability to liquid water results in more water flowing through the pores, producing less freezing and lower freezing strains. Figure 18b–d show the comparison between the freezing strain of the selected values of liquid water permeability with respect to different amounts of chloride. It can be observed that by increasing the amount of chloride, the freezing strain of PI cement paste for specified liquid water permeability values decreases. Moreover, the influence of permeability decreases with an increase in chloride content.

**Figure 19:**
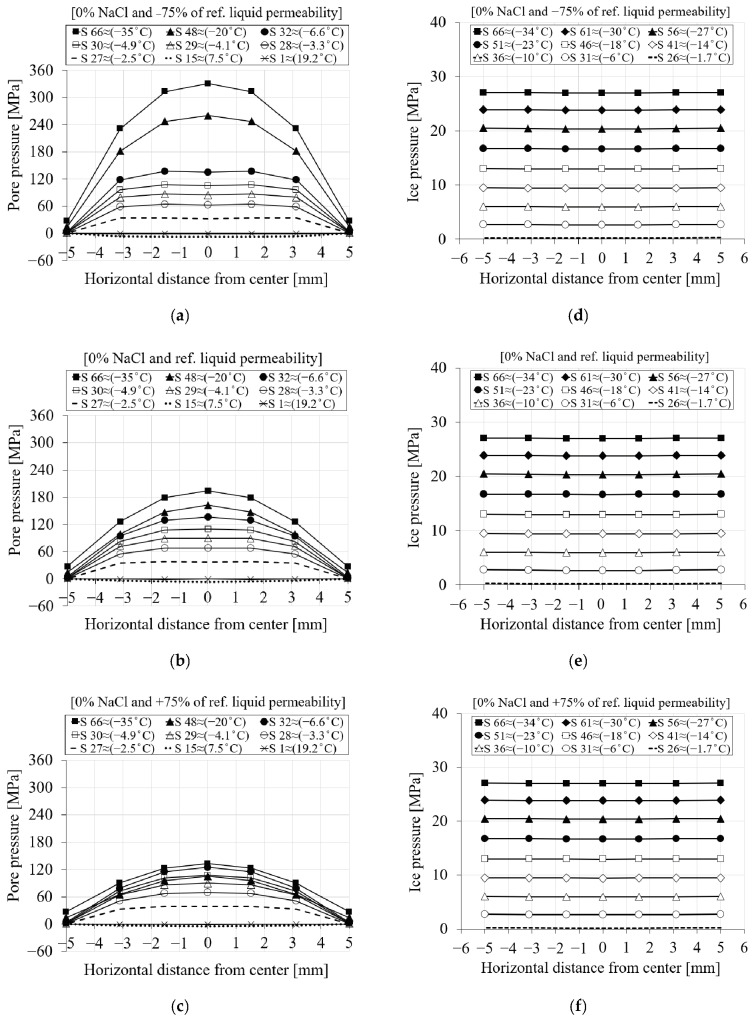
The comparison of the pore and ice pressure of seven selected nodes in terms of distance and time step between the reference and the two extreme liquid water permeability values for PI cement paste with 0% chloride concentration during the first cooling phase: (**a**–**c**) pore pressure; (**d**–**f**) ice pressure.

To better illustrate how changes in liquid water permeability affect the induced pore and ice pressure, the pore and ice pressure for the first cooling phase are shown as a function of freezing time and distance in Figure 19. Only the results for two extreme values of liquid water permeability compared to the reference value for 0% chloride concentration are shown. As mentioned earlier, the positive pore pressure starts to develop as soon as the temperature drops below the freezing point of the solution and increases until all pores are frozen.

Figure 19a–c show the pore pressure for −75%, reference and +75% liquid water permeability. From Figure 19a, it can be seen that as the value of liquid water permeability decreases, less solution penetrates through the pores as freezing begins, and therefore more expansion occurs, resulting in higher pore pressure. In contrast to lower liquid water permeability (Figure 19a), Figure 19c shows the pore pressure results for the same time steps for a higher liquid water permeability compared to the reference value. As can be seen, the pore pressure generated is lower for larger liquid water permeabilities than for the reference.

Figure 19d–f show the ice pressure for the three liquid water permeabilities the same as above. It can be seen that the changes in liquid water permeability have no effect on the induced ice pressure. This is mainly because the ice pressure does not start to grow until the solution in the pores is frozen.

### 4.2. Liquid Water Permeability, Total Porosity and Pore Size Distribution

This parametric study was performed to investigate the effect of simultaneous variations of liquid water permeability, total porosity and pore size distribution on the freezing strain and pore pressure of PI cement paste compared to the reference case. All other material parameters remained constant. This case is more realistic since it considers cement paste to have similar mechanical properties (strength, stiffness) but a different pore structure. As it is clear, porosity in cement paste is influenced by different factors, including the water/cement ratio, curing conditions and the type and amount of cementitious material used in the mixture. Therefore, cement pastes with the same compressive strength may still have different porosities if they were mixed with different amounts of water, cured under different conditions, or depending on the type and amount of cement.

Table 5 shows the three different values of total porosity and their corresponding liquid water permeability values used in this study. For each total porosity value, the corresponding cumulative pore size distribution was derived from the original curve in [9] and shown in Figure 17. It is worth mentioning that the coefficient of thermal expansion as a function of porosity is calculated for each porosity case based on Equation (14) and reported in Table 6.

**Figure 20:**
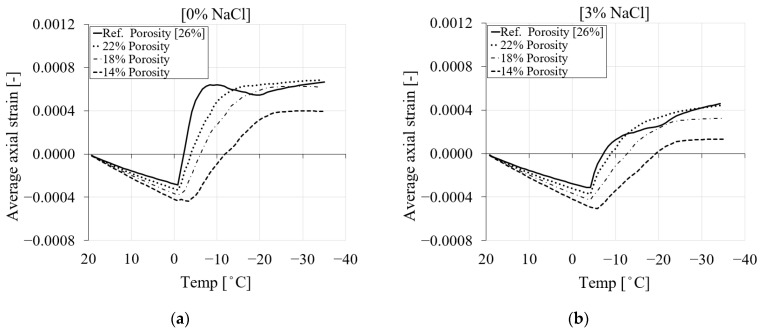

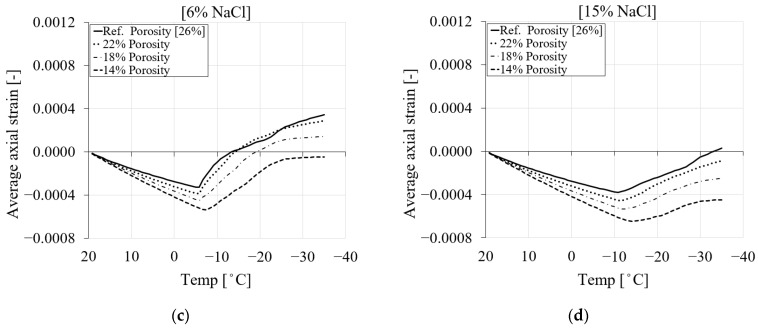
The comparison between the freezing strains of the reference porosity and liquid water permeability and three selected values based on Table 5 for PI cement paste during the first cooling phase: (**a**) pure water; (**b**) 3% NaCl; (**c**) 6% NaCl; (**d**) 15% NaCl.

The numerical results showed that the influence of porosity and liquid water permeability can significantly affect the freezing strain of PI cement paste during the initial cooling stage. A comparison between the freezing strains of the reference case (reference liquid water permeability) and three selected values of porosity and liquid water permeability (Table 5) for four chloride concentrations is shown in Figure 20.

In general, it is shown that when the total porosity is decreased, the material becomes denser and contracts more due to the effect of the thermal expansion coefficient at a decreasing temperature. Consequently, the freezing strain will decrease. The results show that the material with lower total porosity contracts more, and therefore its induced freezing strain (expansion) is smaller than in the case of higher porosity. Figure 20a shows the average axial strain comparison between the reference porosity and the three selected porosity values for pure water. It is visible from this figure that the material with the higher porosity undergoes smaller contractions followed by more expansion. However, the denser material undergoes more contraction, which causes a delay in the expansion phase. The effect of three levels of chloride concentration on the overall deformation of the material for the specified porosity cases is shown in Figure 20b–d. The results show that an increase in the amount of chloride, together with a lower porosity, contributes to more contraction and a delayed phase change. This is related to the fact that the equilibrium pore radius of pores is influenced by both the level of chloride concentration and the presence of smaller pores.

**Figure 21:**
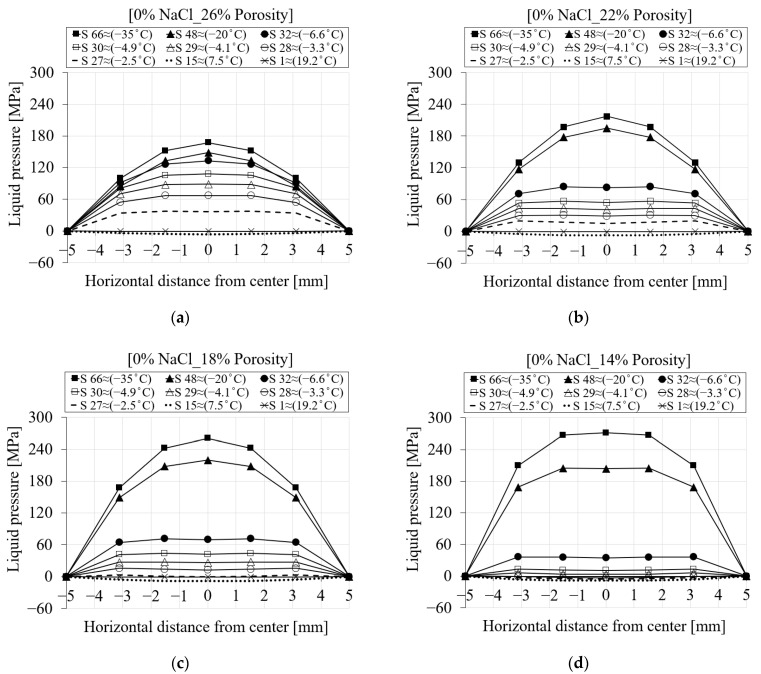
The comparison between the liquid pressure of the reference porosity and liquid water permeability and three selected values based on Table 5 for PI cement paste during the first cooling phase: (**a**) 26% porosity; (**b**) 22% porosity; (**c**) 18% porosity; (**d**) 14% porosity.

The evaluation of the numerical results for the first cooling phase for PI hardened cement paste showed that the modified porosity and the corresponding liquid water permeability influence the liquid and ice pressure generated during the freezing process. It should be noted that a modified porosity volume indicates a different pore structure and behavior of the material. While the relationship between porosity and liquid water permeability is not always straightforward, as other factors can also affect the permeability of the hardened cement paste, in general, lower porosity results in lower liquid water permeability because there are fewer pathways for water to flow through. This phenomenon leads to the buildup of higher fluid pressure compared to a more porous material. Figure 21 shows the liquid pressure for four porosity cases according to Table 5 for pure water. It is evident from Figure 21a that the cement paste with the highest porosity has the lowest liquid pressure. Figure 21b (22% porosity) shows a higher liquid pressure for the selected nodes compared to Figure 21a (26% porosity) from steps 48 to 66. However, for the time steps below step 48, the liquid pressure is higher for the cement paste with 26% porosity compared to Figure 21b. Due to the location of the selected nodes and a completely different pore size distribution between these two cases, a direct comparison of the induced liquid pressure cannot be made, but in general, it is evident from these figures that the material with lower porosity results in a higher liquid pressure. A similar trend can be seen in the other cases.

**Figure 22:**
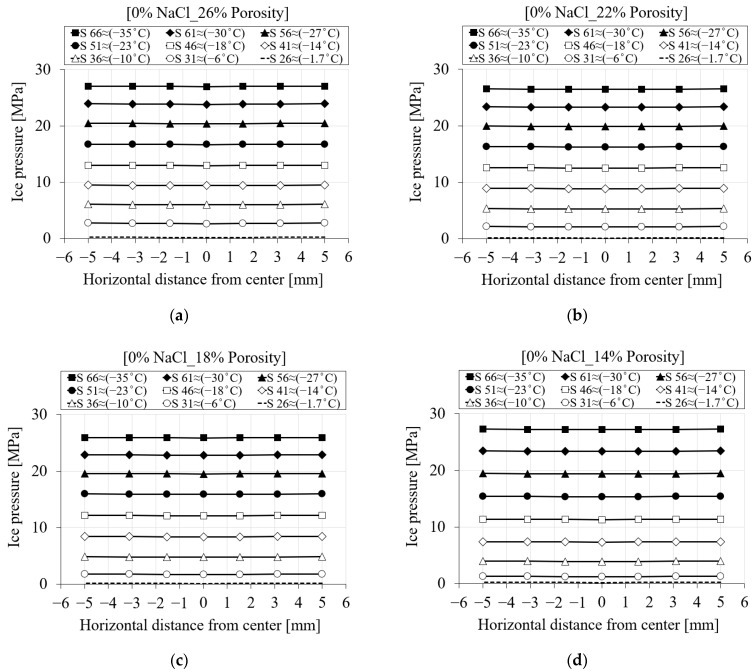
The comparison between the ice pressure of the reference porosity and liquid water permeability and three selected values based on Table 5 for PI cement paste during the first cooling phase: (**a**) 26% porosity; (**b**) 22% porosity; (**c**) 18% porosity; (**d**) 14% porosity.

Figure 22 shows the ice pressure for four porosity cases for pure water. The results in Figure 22a–c show no significant difference in ice pressure, but the ice pressure in Figure 22d is slightly higher for 14% porosity than in the other cases. This is likely related to the higher proportion of smaller pores (Figure 17) compared to the other porosity curves, resulting in less space available for ice expansion during the freezing process, leading to higher pressure.

A comparison of the results of cases 1 and 2 shows that for a fixed overall porosity and pore size distribution, changes in water permeability can significantly affect pore pressure and freezing strain. Lower permeability to liquid water reduces the rate at which water moves through the pores, resulting in higher pressure. However, if one reliably changes the permeability to liquid water and the pore size distribution based on the overall porosity of the material, the cement paste with lower permeability will experience more contraction and less expansion during the freezing process.

## 5. Conclusions

In the current study, the freezing behavior of two saturated cement pastes (PI and PII) with different chloride concentrations was numerically investigated using a coupled 3D hygro-thermo-mechanical FE model implemented in the in-house code MASA. The obtained results can be summarized as follows:

- The FE model can realistically replicate the freezing behavior of the investigated cement pastes in terms of generated pore pressure and consequent material deformation. A good agreement is observed between the here-obtained results and those (numerical and experimental) reported in [9].
- For cement paste with high porosity and water/cement ratio (PI), the deformation shows contraction followed by expansion. However, in the denser cement paste with lower porosity (PII), only contraction is observed due to the denser structure of the paste as well as smaller pore sizes, i.e., no freezing occurred.
- Pore pressure is mainly generated in PI cement paste. The results showed that the maximum pore pressure is observed in the center of the specimen, with a gradual reduction towards the surface of the specimen.
- The chloride concentration in the pore solution has a significant influence on the generated pore pressure and deformation. This is due to a delay in the freezing process.
- Based on the experimental results in [9], the freezing point of the pore solution for the same chloride concentration has been modified in the model, with a consequent delay in the freezing process and better agreement with the experimental deformation curves shown in [9].
- The results of the parametric study showed that for the same amount of chloride concentration, lower liquid water permeability leads to higher pore pressure and deformation, while higher permeability reduces both pore pressure and deformation. However, the generated ice pressure is independent of water permeability.
- The parametric study on liquid water permeability, total porosity and pore size distribution showed that the total porosity and its corresponding liquid water permeability can influence liquid pressure and freezing deformation. However, the results did not show a significant difference in ice pressure. As total porosity decreases, permeability to liquid water decreases, resulting in higher liquid pressure. However, cement pastes with lower porosity showed higher contraction and lower expansion during freezing, which can be attributed to the influence of thermal contraction during the cooling phase.
- Finally, this study has focused on the freezing behavior of cement pastes during the first cooling phase. However, the main challenge in simulating frost action in cementitious materials is related to cyclic freeze–thaw. Understanding the relationship between liquid permeability, porosity and changes in the pore structure during a freeze–thaw attack is essential for accurately predicting the performance of the cement paste under cyclic freeze–thaw loading. This aspect is a perspective on the current work and will be addressed in future investigations. Also, the mesoscale modeling approach will be used to investigate the freeze–thaw behavior of concrete.

## Figures and Tables

**Table 1:** Input parameters for the calculation of equilibrium pore radius, *R_peq_*.

Property	Symbol	Units	Value	Reference Source
Surface tension of the ice/water interface	τ	[N/m]	$\tau =\left(36+0.25\theta \right)\times 10^{3}$	[32]
Mass density of water	*ρ_w_*	[kg/m^3^]	1000	[35]
Mass density of ice	*ρ_i_*	[kg/m^3^]	916	[35]
Fusion entropy density of ice	*Sf*	[J/(K × kg)]	$\frac{S_{f}}{V_{c}\times \rho_{i}}$	[34]
Molar fusion entropy of ice	*S_f_*	[J/(K × mol)]	22	[34]
Molar volume of ice	*V_c_*	[m^3^/mol]	0.0000196	[34]
Contact angle	Θ	[Degree (°)]	Calculated based on chloride concentration in %; see Figure 1a	[34]
Gas constant	*R*	[J/(K × mol)]	8.31441	[34]
Activity of water	*ln_aw_*	[-]	Calculated based on chloride concentration in mol; see Figure 1b	[34]
Unfrozen layer thickness	δ	[nm]	$1.97{\left|\theta \right|}^{-\frac{1}{3}}$	[33]

**Table 3:** Input material properties of porous medium and solid skeleton for PI (PII) cement pastes.

Property	Symbol	Units	Value	Reference Source
Apparent density	*ρ*	[g/mL]	1.6 (2)	[9]
Poisson’s ratio	*ν*	[-]	0.20	[18]
Water/cement ratio	*-*	[-]	0.5 (0.3)	[9]
Total porosity	*n*	[m^3^/m^3^]	0.26 (0.13) Figure 5	[9]
Elastic modulus	*E*	[GPa]	26 (36) *E* = 3(*K*_0_)(1 − 2*ν*)	[9]
Compressibility modulus of the porous skeleton	*K*_0_	[GPa]	14.6 (20.2) *K*_0_ = *E*/3(1 − 2*ν*)	[9]
Compressibility modulus of the solid matrix	*K_m_*	[GPa]	31.8 *K_m_* = *K*_0_/(1 − *n*)^3^	[9]
Biot’s coefficient	*b*	[-]	*b* = 1 − *K*_0_/*K_m_*	[31]
Pore size distribution	*-*	[% by vol]	Figure 5	[9]
Thermal conductivity	*λ_m_*	[W/(m × K)]	1.15 (1.35)	[9]
Heat capacity	*C_m_*	[J/kg × K]	840.0	[35]
Thermal expansion coefficient	*α*	[°C^−1^]	1.20 × 10^−5^ (1.86 × 10^−5^)	[40]
Liquid water permeability	*D*	[m^2^]	2.78 × 10^−21^ (2.29 × 10^−19^)	[2]

**Table 4:** Input material parameters for water and ice.

Property	Symbol	Units	Value	Reference Source
Density of water	*ρ_w_*	[kg/m^3^]	1000	[35]
Water compressibility	*K_w_*	[GPa]	2	[35]
Liquid water coefficient of volumetric thermal expansion	*α_1_*	[°C^−1^]	*α*_1_ = (−9.2 + 2.07*Ө*) ×10^−5^	[18]
Thermal conductivity of water	*λ_w_*	[W/(m × K)]	0.55	[35]
Heat capacity of water	*C_w_*	[kJ/(kg × K)]	4.22	[35]
Dynamic viscosity of water	*η*	[Pa × s]	*η* = 1.38 × 10^−6^ exp(2.590/*T*) with *T* in *K*	[18]
Density of ice	*ρ_i_*	[kg/m^3^]	916	[35]
Ice compressibility	*K_i_*	[GPa]	8	[35]
Ice coefficient of volumetric thermal expansion	*α_i_*	[°C^−1^]	α_i_ = 5.5(1 + (*Ө*/200)) × 10^−5^	[18]
Thermal conductivity of ice	*λ_i_*	[W/(m × K)]	2.20	[35]
Heat capacity of ice	*C_i_*	[kJ/(kg × K)]	2.11	[35]

**Table 6:** Thermal expansion coefficient for four different porosity cases.

**Total Porosity**	**26%** **(Ref. Value)**	**22%**	**18%**	**14%**
Thermal expansion coefficient (°C^−1^)	1.20 × 10^−5^ (ref. value)	1.39 × 10^−5^	1.59 × 10^−5^	1.81 × 10^−5^

**Figure A1:**
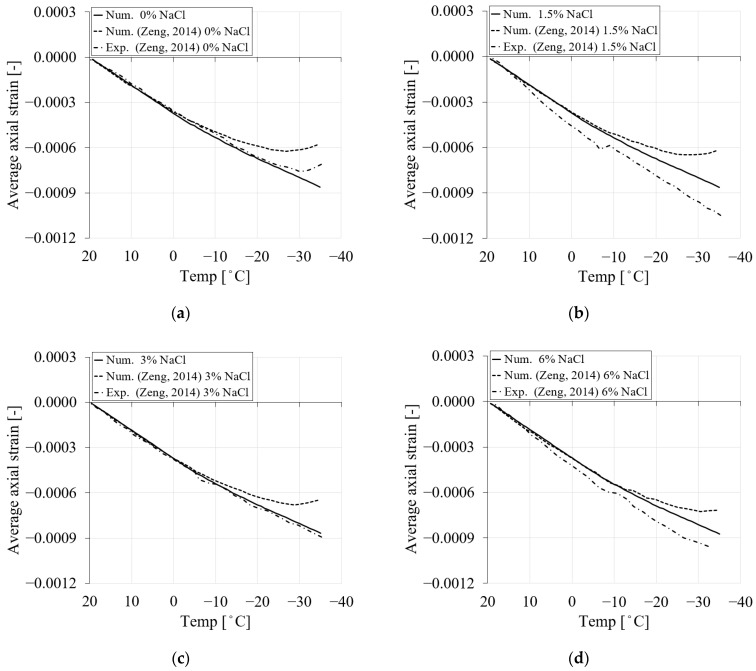

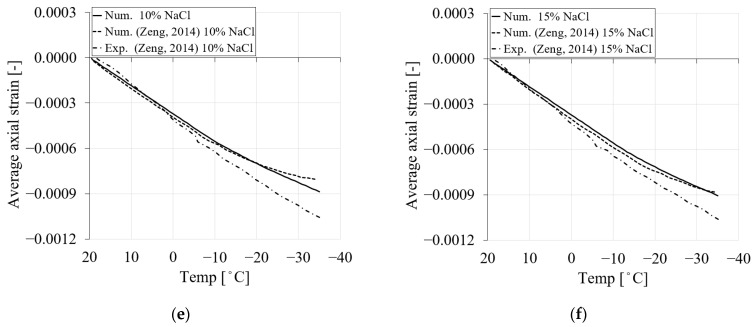
The comparison between the freezing strains of the current study and experimental and numerical results from Zeng [9] for PII cement paste during the first cooling phase: (**a**) pure water; (**b**) 1.5% NaCl; (**c**) 3% NaCl; (**d**) 6% NaCl; (**e**) 10% NaCl; (**f**) 15% NaCl.

## Data Availability

The data presented in this study will be made available upon request.

## Appendix A

### Appendix A.1. Numerical Results and Discussion for PII Cement Paste (0.3 w/c Ratio)

The second set of simulations for PII cement paste with a water/cement ratio of 0.3 (case 2) and chloride concentrations of 0, 1.5, 3, 6, 10 and 15% were simulated, and the resultant average axial strains were compared to those of the experimental and numerical results from [9]. The comparison between the average axial strains measured in the current study and in [9] in terms of freezing strain and freezing temperature for the first cooling phase is shown in Figure A1.

As can be seen, the experimental and numerical results for PII specimens from [9] show very different deformation responses compared to the PI specimens. All specimens showed no visible expansion at below-freezing temperatures and no appreciable residual freezing strain. The denser microstructure of PII material compared to PI cement paste can be the only reason for this difference. The paste includes much fewer capillary pores and has substantially less pore percolation due to its lower *w*/*c* ratio of 0.3. The results also show a complete contraction of the PII specimen and are in very good agreement with the experimental and numerical results shown in [9] for the first cooling phase. The results for liquid, ice and pore pressure are not reported for PII cement paste because, due to the pore structure of the material, no freezing occurred within the capillary pores for PII and, consequently, no pore pressure or related expansion was generated.
